# Incidental Finding of Saddle Pulmonary Embolism on a CT Scan of the Abdomen and Pelvis in a Patient With Adenocarcinoma of the Colon

**DOI:** 10.7759/cureus.20757

**Published:** 2021-12-27

**Authors:** Seyed M Nahidi, Uzayr Ali, Leonidha Duka, Juan C Fuentes-Rosales, Utpal Bhatt

**Affiliations:** 1 Medicine, St. George's University, St. George's, GRD; 2 Medicine, Wyckoff Heights Medical Center, Brooklyn, USA; 3 Internal Medicine, Wyckoff Heights Medical Center, Brooklyn, USA; 4 Pulmonology, Wyckoff Heights Medical Center, Brooklyn, USA

**Keywords:** saddle pulmonary embolism, thromboembolism, incidental finding, xeloda, adenocarcinoma of the gastrointestinal tract

## Abstract

A saddle pulmonary embolism is defined as a large thromboembolus lodged at the bifurcation of the pulmonary artery. It would be expected for a patient with a saddle pulmonary embolism to present with symptoms such as dyspnea or pleuritic pain. However, more often than not, saddle pulmonary embolisms may present asymptomatically and are not associated with the typical symptoms. We present a case of an incidental finding of saddle pulmonary embolism in an 89-year-old patient with a past medical history significant of gastrointestinal adenocarcinoma that was treated with capecitabine. The saddle pulmonary embolism was found incidentally on computer tomography (CT) with the contrast of the abdomen and subsequently confirmed with CT of the chest with contrast. It is crucial to be mindful of a possible pulmonary embolism in a patient with similar past medical history.

## Introduction

A saddle pulmonary embolism is a thromboembolus that causes a mechanical obstruction in the bifurcation of the pulmonary artery. This can lead to a wide variety of symptoms such as dyspnea, pleuritic chest pain, or a cough. However, it is not uncommon for a patient to have asymptomatic saddle embolism or have a non-specific symptom. The reason why a patient with saddle embolism remains asymptomatic is still unknown. Possible mechanisms may include the development of collateral vessels to allow a normal amount of blood flow to the lungs or that the embolus was not large enough to cause any symptoms [1]. Patients with risk factors, especially adenocarcinoma of the gastrointestinal tract treated with capecitabine, may be at a greater risk for the development of a pulmonary embolism or a saddle embolism. We present an incidental finding of a saddle pulmonary embolism in a patient with adenocarcinoma of the ascending colon treated with capecitabine.

## Case presentation

An eighty-nine-year-old male presented to our institution with non-bloody non-bilious vomiting, nausea, cough with white sputum, weakness, and poor oral intake. He also complained of mild epigastric pain. His past medical history was significant of hypertension, benign prostatic hyperplasia, hard of hearing, and stage 3Bp T3 N1 adenocarcinoma of the ascending colon status-post right hemicolectomy followed by adjuvant capecitabine, which was completed about two months prior to this current admission. As per the next of kin (NOK), he had no other symptoms, including headache, dizziness, chest pain, shortness of breath, palpitations, fever, chills, and any recent falls/trauma.

Upon admission, the patient had a temperature of 96.3 F (35.7 C), heart rate of 61/min, respiration rate of 20/min, O2 saturation of 98% on room air, and blood pressure of 141/75 mmHg. As per next of kin, the patient only takes aspirin at home. The patient was alert and oriented to person and place, which was the baseline. He appeared to be malnourished. The patient was in no acute distress, lungs were clear to auscultation, the heart was in regular sinus rhythm, and normal heart sounds were auscultated. The abdomen was non-distended but mildly tender to palpation in the epigastric area. The patient had no organomegaly. His initial troponin was 0.056 ng/mL, and lactic acid was 2.2 mmol/L. He was started on a one-liter lactate ringer fluid. He tested negative for Covid-19. The patient was an ex-smoker and denied alcohol and recreational drug use.

**Figure 1:**
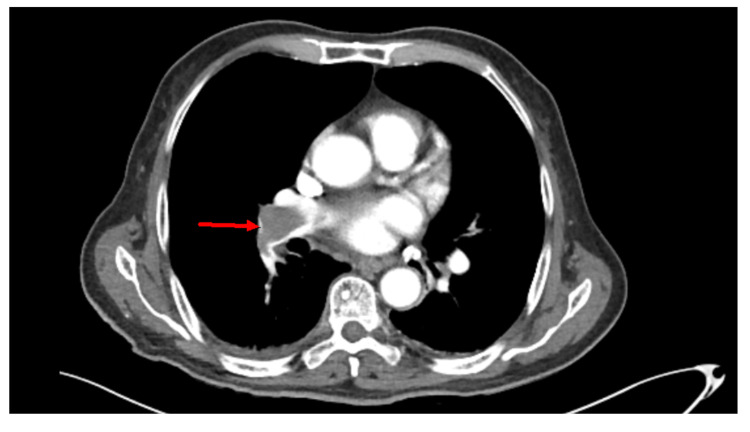
Incidental finding of a saddle embolism on an abdominal and pelvis CT scan

**Figure 2:**
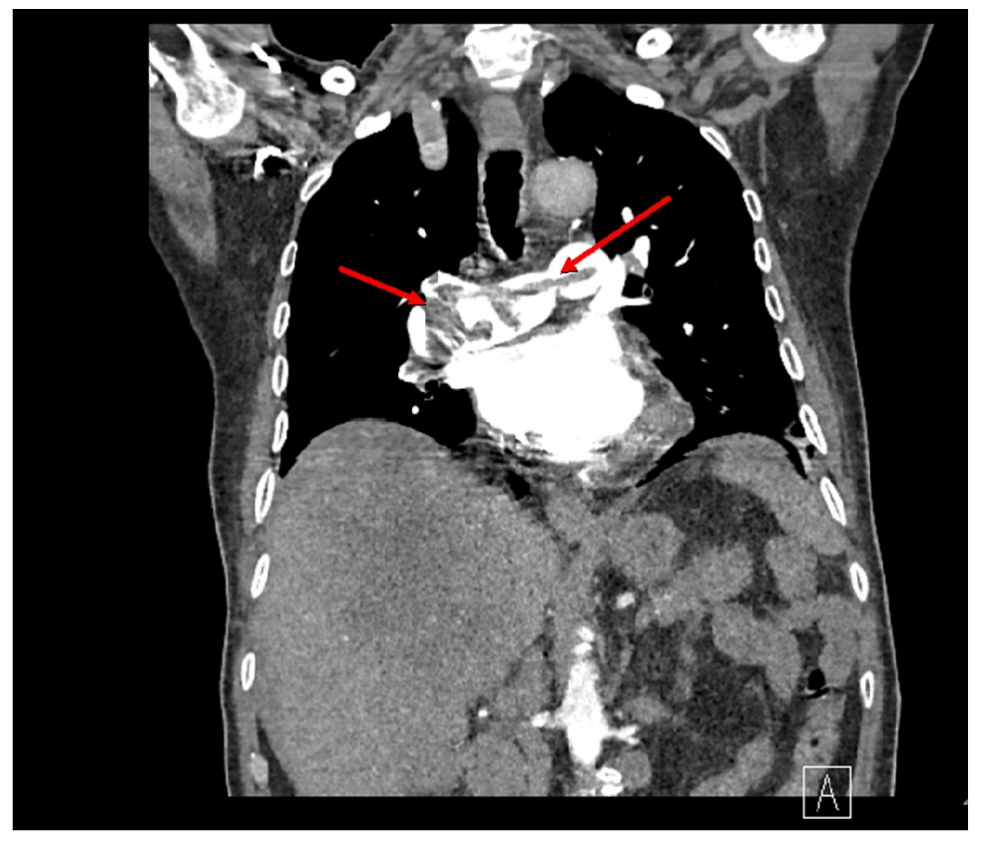
Axial CT scan of the chest for confirmation of the saddle pulmonary embolism

**Figure 3:**
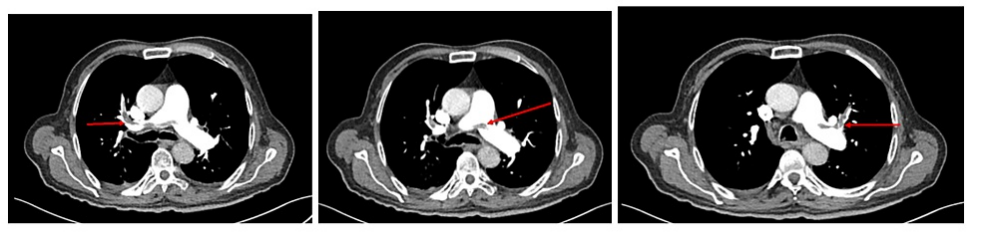
Axial CT scan of the chest for confirmation of the saddle pulmonary embolism

The chest X-ray showed no acute cardiopulmonary disease. CT of the abdomen and pelvis with contrast identified a mass, 12 by 12 cm, in the liver, lymphadenopathy, abdominal nodularity, and bilateral pulmonary embolism (Figure 1). The liver mass identified on the CT scan of the abdomen and pelvis suggested severe hepatic metastatic disease. A CT of the chest was ordered that confirmed the presence of a saddle pulmonary embolism (Figures 2, 3). The pulmonologist recommended a venous doppler ultrasound of the lower extremity, an echocardiogram to assess for right heart strain due to his age, and an electrocardiogram. The venous doppler ultrasound of the extremities was negative. The echocardiogram identified an ejection fraction of 50-55%, basal to mid-portion of the inferior wall was hypokinetic, the apical lateral wall was hypokinetic and moderate aortic regurgitation. There was no evidence of right heart strain. The patient was found to have a prolonged QT interval on EKG (corrected QT interval [QTc] of 490 ms) and was given calcium carbonate. His second troponin was 0.052, and his second lactic acid was 1.8. The patient was given a proton pump inhibitor and placed on a heparin drip. 

Nausea, vomiting, and cough improved during the hospital stay. The nausea and vomiting were attributed to gastroesophageal reflux disease (GERD) vs. hepatic metastasis. The cough was attributed to a change in season vs. upper respiratory infection vs. GERD as the patient did not have a fever or an elevated white blood cell count. The patient was placed on Eliquis 10 mg twice a day (BID) for the first seven days and 5 mg BID for the next 14 days as per the hematology-oncology specialist. 

## Discussion

A saddle pulmonary embolism (PE) is defined as when a clot is lodged at the bifurcation of the main pulmonary artery. It was thought that saddle PE would inevitably lead to hemodynamic instability and death; however, through retrospective studies, it was found that only 22% of those diagnosed with a saddle PE are hemodynamically unstable with only a 5% mortality rate [2,3]. There is also a 10% increase in the risk of PE in patients with malignancy [4,5]. Other associated risk factors are age ≥65, male sex, metastatic disease, ascites, congestive heart failure, BMI ≥25 kg/m2, platelet count >400,000/microL, serum albumin <3 g/dL, and duration of surgery >2 hours [6]. Only three of which were present in our patient. 

In a normal course of any PE, the patient would be expected to present with dyspnea (73%), pleuritic pain (66%), cough (37%), orthopnea (28%), calf pain (44%), wheezing (21%), or hemoptysis (13%) [7]. However, our patient only presented with a cough which, in a clinical setting, does not draw attention to a possible PE. The use of troponin levels was the primary clue in identifying the presence of a PE, as troponin levels are raised in 30-50% of patients with larger pulmonary emboli [8,9]. This transient increase in troponin resolves within 40 hours as compared to the risk associated with an acute myocardial infarction [10]. 

Asymptomatic PE may occur in up to 5% of patients with carcinomas, including those of the gastrointestinal tract [11]. However, in patients with symptomatic saddle embolism, an echocardiogram report may reveal a right ventricular enlargement and/or dysfunction. However, our patient’s echocardiogram did not reveal such findings. A possible reason why our patient was hemodynamically stable may be due to the emboli not obstructing more than 75% of the normal volume of flow through the artery and subsequent pulmonary vasculature [12]. This allows for the right ventricle to not exceed its normal pressures, maintaining hemodynamic stability. This can be further supported by the fact that only four in 48 patients, post-surgery, presented symptomatically with PEs in one study [13].

Once a diagnosis of saddle PE is made, the mainstay of treatment is anticoagulation. Adenocarcinoma of the GI tract, as seen in our patient, is known to be associated with greater hypercoagulability than other forms of neoplasms [14]. A study showed that colon cancers are associated with 76 deep vein thrombosis and pulmonary embolism (DVT/PE) per 100,000 patients, with adenocarcinomas being the most common histological finding [15]. This further increases the recurrence rate for PEs and other venous-thrombo-emboli phenomena above normal rates of other malignancies. This increase can be attributed to increased tissue factor, the formation of cancer procoagulant (a calcium-dependent cysteine protease), the presence of increased cytokines due to malignancy, and possible expression of the MET oncogene [16]. Furthermore, our patient was treated with capecitabine, and studies have shown that capecitabine increases the risk of venous thromboembolism [17]. A study conveyed that the overall rate of a venous thromboembolic event in patients with capecitabine is 10.5% [17]. Compounded onto that, the risk of death from PE, with cancer treatment, is the leading cause of death secondary to the risk of death from cancer itself [18]. 

## Conclusions

Asymptomatic pulmonary saddle embolism is more likely not to have any evidence of hemodynamic instability and may even present with very nonspecific symptoms. In cases where a patient with a history of adenocarcinoma of the GI tract with adjuvant therapy with capecitabine, it is imperative to be mindful of the possible silent pulmonary embolisms and VTEs that are associated with these conditions. Early intervention may lead to better outcomes in patient management, lowering potential comorbidities such as progression of the thromboembolism.
